# Bio-Based Rigid Polyurethane Foam Composites Reinforced with Bleached Curauá Fiber

**DOI:** 10.3390/ijms222011203

**Published:** 2021-10-18

**Authors:** Sylwia Członka, Eduardo Fischer Kerche, Roberta Motta Neves, Anna Strąkowska, Krzysztof Strzelec

**Affiliations:** 1 Faculty of Chemistry, Institute of Polymer and Dye Technology, Lodz University of Technology, Stefanowskiego 12/16, 90-924 Lodz, Poland; anna.strakowska@p.lodz.pl (A.S.); krzysztof.strzelec@p.lodz.pl (K.S.); 2 PPGE3M, Federal University of Rio Grande do Sul—UFRGS, Av. Bento Gonçalves 9500, Porto Alegre 91501-970, Brazil; eduardo.fkerche@gmail.com (E.F.K.); robertamneves@gmail.com (R.M.N.)

**Keywords:** rigid polyurethane foams, cellular composite, bleached Curauá fiber, high-performance foams

## Abstract

This study aims to evaluate the influence of using a bleached Curauá fiber (CF) as filler in a novel rigid polyurethane foam (RPUF) composite. The influence of 0.1, 0.5 and 1 wt.% of the reinforcements on the processing characteristics, cellular structure, mechanical, dynamic-mechanical, thermal, and flame behaviors were assessed and discussed for RPUF freely expanded. The results showed that the use of 0.5 wt.% of CF resulted in RPUF with smoother cell structure with low differences on the processing times and viscosity for the filled pre-polyol. These morphological features were responsible for the gains in mechanical properties, in both parallel and perpendicular rise directions, and better viscoelastic characteristics. Despite the gains, higher thermal conductivity and lower flammability were reported for the developed RPUF composites, related to the high content of cellulose and hemicellulose on the bleached CF chemical composition. This work shows the possibility of using a Brazilian vegetable fiber, with low exploration for the manufacturing of composite materials with improved properties. The developed RPUF presents high applicability as enhanced cores for the manufacturing of structural sandwich panels, mainly used in civil, aircraft, and marine industries.

## 1. Introduction

Bio-based filled rigid polyurethane foams (RPUF) are a new class of polymer composite materials that present interesting properties. Some recent studies on the field proved that when a vegetable fiber, or a filler, is introduced, as a second phase, the cellular composite presents increases of properties, such as thermal stability, fire resistance, and photodegradation performance [1,2,3,4,5,6]. Technological properties, as decreases of thermal conductivity, water/moisture uptake, greater dimensional stability, and aesthetical appearance, antibacterial/anti-aging properties are also achieved [7,8,9,10,11,12,13,14]. Moreover, bio-based polyols can be used, instead of petrochemical ones, as feedstock, aiming to produce environmentally-friendly RPUF [15,16,17,18]. However, to improve the mechanical properties of RPUF, to designate the material as a structural component, some issues need to be taken into account, as the filler’s type/aspect ratio, as well as its compatibility with the PU matrix [19].

It was shown in previous reports that these fillers characteristics influence directly the foaming process and foams’ curing kinetics, which affects directly its cellular foams’ morphology (cells’ anisotropy, size, distribution, and closed cells content) and consequently on the final foam’s performance [9,20]. In this sense, some strategies have been developed to improve mechanical properties of filled-RPUF, as the chemical surface treatment of fillers, by the use of silanes, maleic anhydride, alkali treatments [2,21,22], aminopropylisobutyl-polyhedral oligomeric silsesquioxanes [23], ionic liquids [24,25,26], plant oils [27], and by the incorporation of combined fillers [8,28].

Bleaching is another and cheaper surface pre-treatment that can be beneficial for the production of vegetable reinforcements. By the combination of sodium-based salts and peroxides, fibers with lower polymerization degree, extractives, and lignin contents, as well as higher aspect ratio, cellulose, and hemicellulose contents are expected [29]. Furthermore, bleaching pre-treatment may be beneficial for the production of micro or nano-fibrillated cellulose, due to the lower tendency to form aggregates, when the milling process is applied [30,31]. The reduction of particles’ surface free energy, promoted by surface treatments, may decrease the agglomeration of fillers when they are dispersed into the polyol. These clusters can actuate as a discontinuity for the cells’ formation, and then rupture the cells’ edges during the foams’ rising, reducing the foams’ performance [13,30,31,32].

Fibrillated fillers are also more interesting alternatives when improvements on mechanical properties (compressive, flexural, or tensile) and viscoelastic characteristics are desired [31,33,34]. Due to the alignment of the fibrils, by shear forces, when the foam is rising, improved mechanical properties parallel to the rise direction are expected [35]. Besides, thinner cells with consequent decreases of cells’ diameter and increases of cells’ anisotropy are expected, due to the action of such fillers as nucleating agents for the bubbles’ formation, which, in turn, influence apparent density and thermal conductivity [32].

Tough fibrillated fillers, like glass fibers, may also be used to reinforce RPUF, promoting dramatic improvement in its mechanical performance. Structural components, like those used in the liquefied natural gas industry and when the component is destined for core bumpers fabrication, used in the automotive industry, are some applications’ examples of such components [36,37]. However, synthetic fillers present some drawbacks, as the use of non-renewable materials and the high consumption of energy for reinforcement production. In this sense, vegetable fibers, like sisal, jute, ramie, kenaf, coconut, flax, and Curauá, become a greener alternative to substitute synthetic fillers. Moreover, the production of vegetable fibers is associated with social and economic developments of less favored regions, where the reinforcements are commonly cultivated [38].

Specifically, Curauá (Ananas erectifolius) fiber (CF) is highlighted among the vegetable fibers. Depending on the CF crop, it is expected that its stiffness may reach 5–9 times greater than sisal or jute, also presenting lower density among the aforementioned fibers [39]. Interestingly, Curauá in nature is constituted of 70–74% of cellulose, which responsible for the high strength and stiffness of vegetable fibers [40]. According to Satyanarayana et al. [39], CF can reach tensile strength ≈3 GPa and modulus ≈80 GPa, with a density 30 times lower than E-glass, depending on the fiber diameter and source. These characteristics make CF a good candidate to substitute glass fibers in many applications, aiming to produce environmentally friendly composites [30,41].

As presented, CF has outstanding physical-mechanical properties. Due to it, they have been applied as reinforcement for non-porous composites [42,43]. To the best of our knowledge, there are no attempts reported in the literature reinforcing RPUF by bleached CF. Apart from it, the novelty of this paper is the manufacturing and characterization of RPUF/CF. Different contents of CF were used and a deep discussion, relating the morphological aspects, when the CF content is varied, with RPUF properties is presented, aiming to diffuse and extend the applications of CF for polymer composite materials.

## 2. Results and Discussion

**Figure 1:**
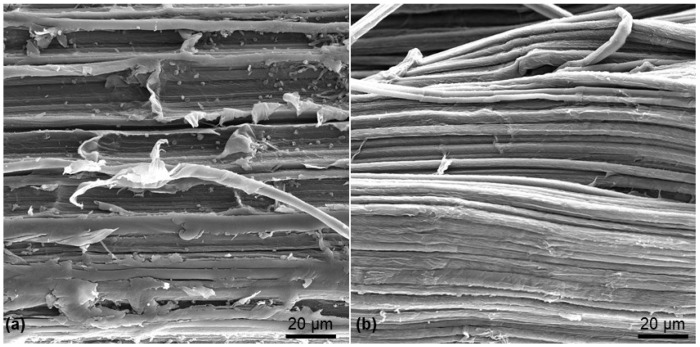
Unbleached CF (**a**) and bleached CF (**b**).

Figure 1 presents the SEM images for the unbleached (UCF) and bleached Curauá fibers (BCF). It is possible to see that the treatment removed a large amount of dirty and constituents of the fibers, like lignin, hemicelluloses, and waxes from the CF surface. Moreover, greater defibrillation is also achieved for BCF, corroborating with other studies of bleached vegetable fibers, like sugar cane and Sisal [44,45,46]. Furthermore, bleaching decreased the polymerization degree (PD) (from 842 ± 13, for UCF to 562 ± 10, for BCF), fiber diameter (from 77.5, for UCF to 4.6 for BCF), and increased the CI (from 70, for UCF to 74, for CF) and increased the cellulose content, as also expected [45,46].

The mean viscosity of each premixed polyols with the specific amount of CF filler is presented in Table 1, as well as the results for the kinetic expansion for the produced RPUF composites. As expected, the greater amount of CF increased the dynamic viscosity significantly, with a higher result for CF 1 [3]. The higher reaction temperature is observed for CF 0.1, which can be related to the high contact between fillers and isocyanate, compared to the other groups.

The great content of hydroxyls on the CF surface can increase the reactivity of the system and also improve the tack-free time for those CF 0.1. However, when a content greater than 0.1 wt.% is employed, the particles’ surface starts to touch each other and, consequently, the contact between fiber-isocyanate decreases, which in turn decreases the reaction temperature and increases cream and total expansion times.

**Figure 2:**
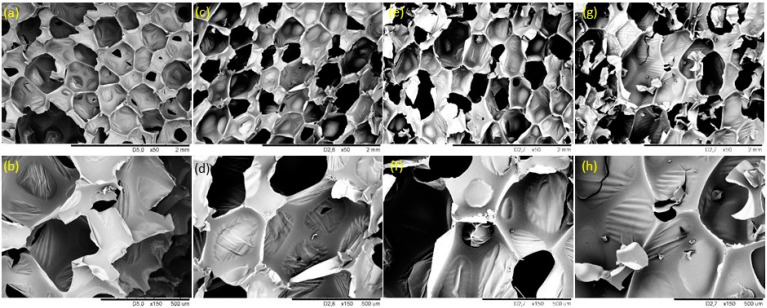
SEM images of the studied RPUF composites: (**a**,**b**) Neat RPUF; (**c**,**d**) CF 0.1; (**e**,**f**) CF 0.5; (**g**,**h**) CF 1.

Figure 2 presents SEM images for all studied RPUF composites. Different magnifications are used, aiming to evaluate the RPUF’s overall aspect and the cells’ aspects. In a general way, a rectangular cell structure is presented for all RPUF composites, as also observed in previous reports [28]. Some factors, as pre-polyol viscosity, filler concentration/interaction, and dispersion influence directly the regularity of the cells’ structure [35]. For that neat RPUF a regular cell structure is observed with an apparent low content of open cells. The number of cells increases when CF is used. However, for the foams with a CF amount greater than 0.1 wt.%, an evident higher number of ruptured cells is observed (Figure 2e–h). Closed cells’ content is presented below and related to these morphological features.

**Figure 3:**
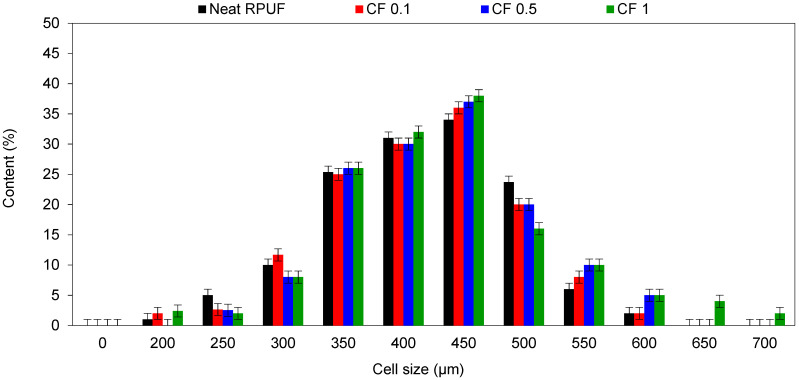
Cell size distribution for the studied RPUF composites.

Figure 3 presents the cells’ size distribution for the studied RPUF composites. In a general way, the use of CF shifts the cells’ distribution to higher values. Although fillers generally act as nucleating agents and, consequently, shift the cells’ distribution to lower ranges [28], fibrillated fillers seem to behave somewhat differently from particle one [24]. Due to the alignment of the fibers, when RPUF is rising, cells’ elongations are expected, especially when the filler is used above the matrix saturation (i.e., those CF 1). Furthermore, the changing of the cells’ mechanism formation, from homogenous to heterogonous, contribute to the overall distribution and lower uniformity of the RPUF cells’ structure [47], highlighting, again, those CF 1.

Table 2 presents the results of apparent density and calculated morphological characteristics. Significant differences for cell size are presented for the RPUF with a higher amount of CF (i.e., 0.5 and 1.0 wt.%). A linear relation between apparent density and cell size is evident, for all RPUF composites. Similar results were reported in previous works [8,28], and are related to the increasing dynamic viscosity of RPUF systems, containing solid particles. Moreover, due to the high affinity of such fillers with PU, they are able to act as nucleation agents for the bubble’s formation, which, in turn increases the number of cells and decreases the apparent density [31]. This also affects the value of R constant which is related to the cells’ elongation. When compared with Neat RPUF, the addition of CF increases the value of R and this effect is more prominent for those CF 1.

**Figure 4:**
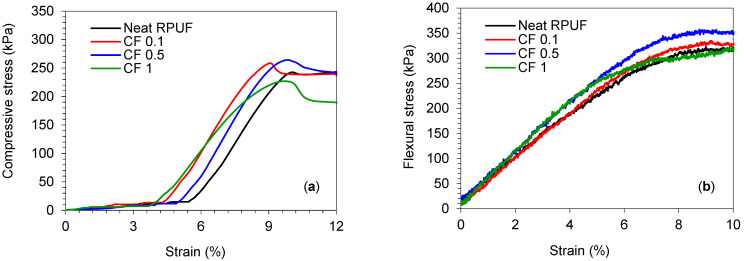
Median curves for compressive (**a**) and flexural (**b**) tests parallel to the rise direction of the studied RPUF composites.

Figure 4 presents the median curves for the compressive parallel and flexural mechanical tests of the studied RPUF composites. Typical elastic–plastic behavior is presented for the compressive tests (Figure 4a) and it is in agreement for stiff RPUF [31]. After sample accommodation (nearly 6% strain), the load increases linearly with strain up to a peak that depends on anisotropy and cell size [48]. After that, the cells start to collapse with each other and microcracks start to take place, which is ascribed as an abrupt decrease in the stress (i.e., close to 10% of strain). The RPUF reinforced by CF, up to 0.5 wt.%, presented a higher apparent modulus compared to the neat one, which is related to the stiffer cell wall of such composites. On the other hand, those CF 1 presented a lower apparent modulus, which may be related to the poorer and irregular cells’ formation, with a greater cells’ edge rupture compared to the others.

For the flexural tests (Figure 4b), all RPUF composites presented a higher apparent modulus (the linear region on the stress–strain curve) compared to the neat foam. For the flexural tests, issues as cell size, distribution, and anisotropy seem to influence minor significance [28]. However, filler saturation and agglomeration represent a great influence for this property, as presented below.

Table 3 presents the calculated results for the RPUF’s mechanical properties. Significant increments for compressive strength parallel/perpendicular and flexural strength are observed when the RPUF are reinforced with CF up to 0.5 wt.%. The latter with ≈9% increment for all calculated mechanical strengths. Until this amount of reinforcement, the saturation of the filler is not reached on the PU matrix, then, the cells are formed without significant irregularity, as presented on SEM images (see Figure 2). Moreover, CF alignment, when RPUF is rising takes place, as aforementioned, which also influences the mechanical properties improvement. On the other hand, those CF 1 presented a significant reduction in all properties, also with an improvement on the maximum elongation at the flexural strength. These results are probably related to the morphological features and filler saturation on the PU matrix, as discussed above.

As presented at [31] and discussed by Hamilton et al. [49], the evaluation of specific properties (i.e., when the mechanical property is divided by the density), for foamy composites is not a realistic evaluation of the influence of reinforcements. Morphological characteristics, due to the high influence of fillers on rising, as cells’ anisotropy and distribution, need to be taken into account. Then, aiming to evaluate the real influence of the CF on the mechanical properties, reinforcement efficiency factors (Γ) were calculated for both compressive and flexural tests (Table 3).

Despite those CF 0.5 presented greater results for mechanical properties, no significant differences for ΓCparallel/perpendicular or ΓF are reported, when they are compared with CF 0.1. Indeed, no significant differences are reported for rheological properties and processing times for both groups, which supports the hypothesis that even in low amounts, CF can reinforce the RPUF cell walls, due to its high aspect ratio and high amount of cellulose/hemicellulose. However, when a high agglomeration of such reinforcements takes place, the fillers start to touch themselves and the cells’ rupture mechanism takes place (as described above), which significantly reduces the values of Γ, for CF 1.

**Figure 5:**
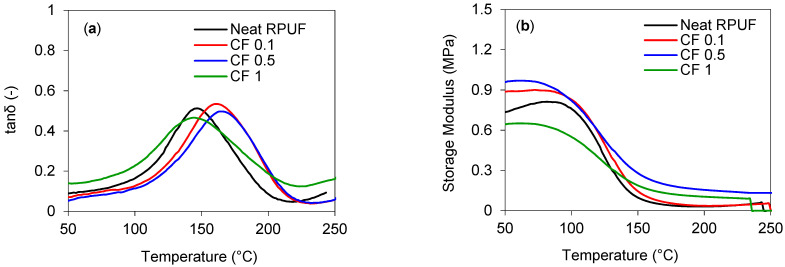
Dynamic mechanical analysis curves for the studied RPUF composites—(**a**) tan δ and (**b**) storage modulus.

Figure 5 presents the DMA curves for all RPUF composites and Table 4 summarizes the main results. Polymers are viscoelastic materials and exhibit three main regions: glassy, transition, and rubbery. Due to this, storage modulus (E’) in glassy and rubbery will be discussed separately [50,51]. Storage modulus (elastic behavior) vs temperature curves are displayed in Figure 5a and Table 4 summarizes the main values extracted from those curves. All RPUF and its composites show the well-defined glassy region, transition region, and the rubbery plateau, respectively at 50–80 °C, 90–150 °C, and above 150 °C, corroborating with [23,52].

The results presented in Table 4 indicates that CF 0.1 and CF 0.5 increased 20.6% and 30.4%, respectively, E′g, compared to the neat RPUF. In other words, the incorporation of CF in lower contents improved stiffness, since the incorporation of rigid fillers, such as CF into a polymeric material may change the chain packaging at a glassy state (below Tg). The results corroborate with Kerche, et al., when they analyzed the influence of MFC onto RPUF [31] and Ye, et al. when they filled RPUF with decabrominated diphenyl ethane and expandable graphite [53]. On the other hand, the incorporation of CF at 1.0 wt.% decreased around 12% E′g, compared to the neat RPUF, probably due to the poor dispersion of higher contents of CF. Additionally, this result can be caused by a weaker interaction between the PU matrix and CF. This behavior was previous observed in [52], when RPUF were reinforced with cellulose nanocrystal and by industrial potato protein in [33].

Regarding E’r, the trend among the samples changed, compared to the E’g values, and all the composites showed higher values, compared to the neat RPUF. Once again, CF 0.5 showed higher values, comparing all composites, and increased more than 400% the value, compared to the matrix. It suggests the effect of the CF is pronounced in rubbery, as explained by Neves et al. [50]. A similar trend was also reported in [31,54,55]. At the transition region, a drop on the curves is reported, characteristic of polymeric materials, and may be related to the reinforcement Effectiveness Coefficient (C), (Equation (2)). From Table 4, it is observed lower C values for CF 1.0 and CF 0.5, respectively. These results indicate that, in the aforementioned samples, a smaller difference between E′g and E′r was achieved, which confirms the high effectiveness of CF.

Tan delta measures the material’s viscoelastic properties and from that, it is possible to determine the full width at half maximum (FWHM), which relates to the system homogeneity [50]. From tan delta curves (Figure 5b) and Table 4, it is possible to observe that there were no significant changes, among the composites, regarding peak height. On the other hand, Tg values increased around 16 °C and 19 °C, for CF 0.1 and 0.5 samples, respectively, compared to the neat RPUF. Similar behavior was also reported in [53]. The incorporation of CF at 0.1 and 0.5 wt.% also causes a broad peak compared to the neat RPUF. According to Czlonka et al. [56], this may be related to the different relaxation mechanisms of the filler and matrix, characteristic of RPUF composites.

**Figure 6:**
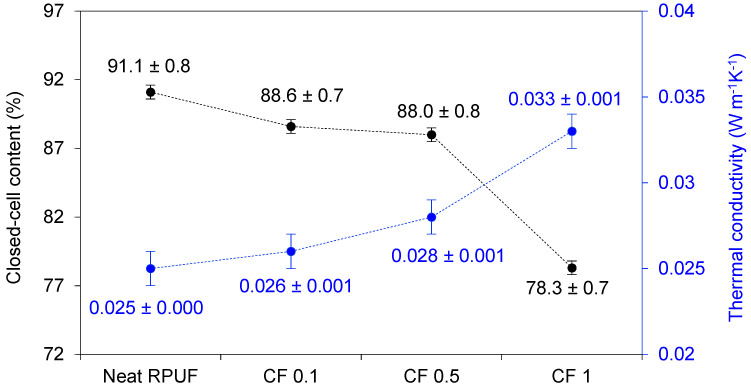
Closed-cell content and thermal conductivity relation for the studied RPUF composites.

Figure 6 presents the results for closed cell content and thermal conductivity coefficient (λ) for the studied RPUF composites. A linear relation between closed cells’ content and λ is reported and they are in agreement with previous reports [2,8,9,33]. Briefly, λ values depend on heat transfer of a solid phase, gas-phase, and convection across the voids. Then, if a higher number of ruptured cells edges are observed, lower values for closed-cell content are expected, corroborating to higher convection of gas through the voids and higher values for λ, as presented here. Besides, the diffusion of CO_2_ through the RPUF cell walls (and replace to atmospheric air) is the main reason for the overall increases of λ (CO_2_ = 0.014 Wm^−1^ K^−1^; air = 0.025 Wm^−1^ K^−1^), and higher values for λ are expected for RPUF that was post-cured for at least 7 days, as the studied one. Finally, the results are in agreement with the SEM images (see Figure 2), where the filled foams presented a more irregular cell structure and consequently lower values for closed-cell contents.

**Figure 7:**
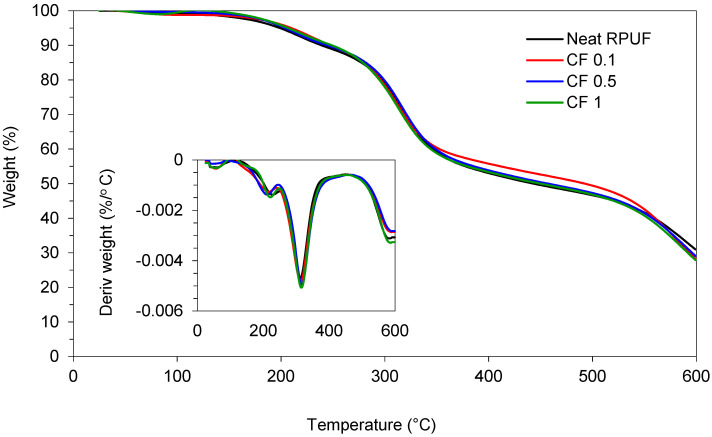
Thermogravimetric curves for the studied RPUF composites.

Figure 7 presents the TGA/DTG results for the studied RPUF composites and Table 5 the main events related to the thermal decomposition of such foams. The results for the 1st stage of thermal decomposition (T_max1_) are related to the low-molecular-weight compounds dissociation, as polyurethane hard segments [57]. Lower values for T_max1_ are expected for filled RPUF and are related to the poor dispersion and aggregates formation into the PU system, which, consequently, changes the RPUF’s cross-link density [3]. At the 2nd degradation stage (T_max2_), fillers decomposition, urea hard segments and higher-molecular-weight compounds (as soft segments) dissociate. A slight increment is expected for filled RPUF, due to the partial linkage of reinforcements to the soft PU-matrix segments [31,57], and also due to the high amount of cellulose of CF.

The last step for thermal decomposition (T_max3_) is related to the decomposition of cellulose and hemicellulose, from vegetable fibers when used in RPUF [23,58]. Higher values for T_max3_ are expected, when a higher amount of CF is used into the RPUF, due to the high content of cellulose of such reinforcements [45]. Finally, a slight decrease in the amount of residue (at 600 °C) is reported for the RPUF with higher contents of CF, related, again, to the high amount of cellulose and hemicellulose on the CF chemical structure. These features facilitate the conversion of polyurethane to volatile gases.

**Figure 8:**
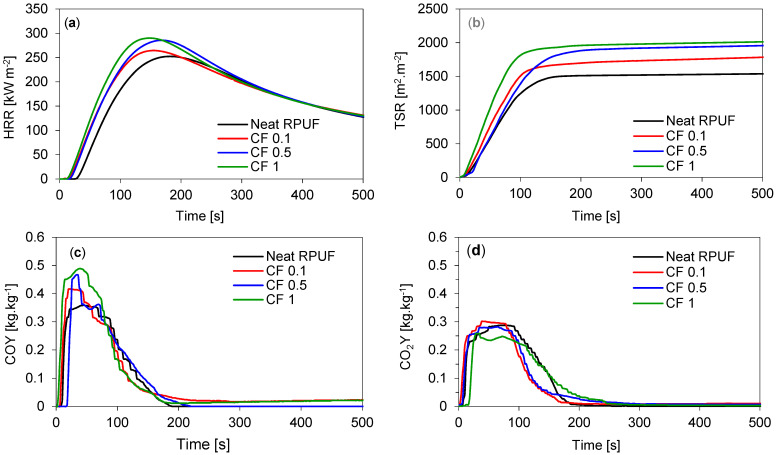
Results from cone calorimetry tests for the studied RPUF composites. (**a**) Heat release rate curve (HRR), (**b**) total smoke release curve (TSR), (**c**) average yield of CO, and (**d**) average yield of CO_2_.

Table 6 presents the compiled results from cone calorimetry for the studied RPUF composites and Figure 8 presents the results of cone calorimetry. It is possible to observe that the ignition time (IT) presented a slight decrease by the use of CF in a content greater than 0.5 wt.%, but no differences were reported for those CF 0.1. This behavior may be related to the high content of cellulose on the CF chemical composition, which has high flammability. Heat peak release (pHRR) is related to the release of low molecular weight compounds, for RPUF, such as isocyanate, olefins, and amines [28]. All RPUF composites presented higher results for pHRR, which may be related to the higher flammability of the foams. However, when the results for the total amount of CO_2_Y are evaluated, lower values are observed as well as the results for limiting oxygen index (LOI).

In a general way, as higher the value of COY/CO_2_Y ratio higher is the incomplete combustion of RPUF and greater amount of toxic smoke is released. All RPUF composites presented higher values for this ratio. Although the flammability of the reinforced foams was impaired, the results from cone calorimetry are in agreement with other studies. In addition, RPUF/CF composites presented lower flammability and indexes, compared with other reports, and lower pHRR and TSR [20,23,28].

**Figure 9:**
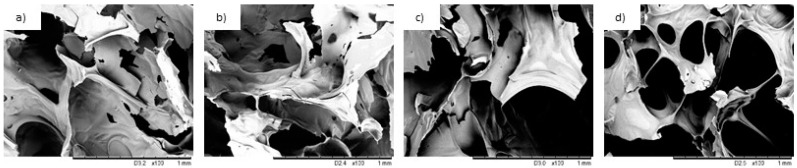
SEM images for the studied RPUF composites residues (after the cone calorimeter test)—(**a**) Neat RPUF; (**b**) CF 0.1; (**c**) CF 0.5; (**d**) CF 1.

Figure 9 presents SEM images of the RPUF residue from the cone calorimetry tests. From the images, it is possible to see a deteriorated cell structure, due to the RPUF burning. Moreover, some burned CF is presented on those foams with high content of the filler, as those CF 0.5 and 1 (Figure 9c,d). As shown in Figure 9a, after the combustion process, the char residue of Neat RPUF presents a compact structure, which may act as a physical barrier against the combustion process. On the other hand, the structure of RPUF composites’ residue seems to be loose and the voids, which are formed during the releasing of the flammable gases are visible in the structure. This effect is most prominent in the case of CF 1. The obtained results indicate that the addition of CF increases the flammability of RPUF due to the biodegradable nature of the filler [6,8,28].

## 3. Materials and Methods

### 3.1. Bleaching of CF and Characterization

The bleaching process of CF was previously described at [45,46]. Briefly, as received CF (UCF) (Support Center for Community Action Projects from Pará state—Brazil) were immersed in 12% (*v*/*v*) NaClO_2_ solution and 5% (*w*/*v*) NaOH solution for 2 h at 50 °C, maintaining 1:10, CF: solution ratio. After this step, the fibers were washed with distilled water until neutralized solution pH, measured by an indicator paper, and oven-dried (Quimis model Q317M) for 12 h at 60°C for further characterization. Scanning Electron Microscopy (SEM, JEOL LTD, Akishima, Japan—conditions described below) was performed for the UCF and BCF, to evaluate the quality of the bleaching process. Other fibers characteristics, as Polymerization degree (PD, determined by viscosity method with the aid of an Ubbelohde viscometer, number 1), crystallinity index (CI), and chemical composition from Fourier transform infrared spectroscopy (FT-IR) and strong acid hydrolysis were previously reported [45,46].

### 3.2. RPUF Preparation and Characterization

The reactant used for the RPUF manufacturing is presented below. All amounts were used in parts by weight (pbw) of the total RPUF mass and the NCO:OH ratio was kept at 160:100. 100.0 pbw—Polyether polyol (Stepanpol PS-2352, Stepan Company (Northfield, IL, USA); 240 mgKOH g^−1^, functionality of 2 and molecular weight of 460 g mol^−1^).

- 160.0 pbw—Polymeric diphenylmethane diisocyanate (MDI) (31% of NCO groups; Purocyn B, Purinova Company; Bydgoszcz, Poland);
- Catalysts: Kosmos 75 (6.0 pbw) (potassium octoate) and Kosmos 33 (0.8 pbw) (potassium acetate) (Evonik Industry; Essen, Germany);
- 2.5 pbw—Silicone-based surfactant: Tegostab B8513 (Evonik Industry (Essen, Germany));
- Blowing agents: (11.0 pbw) Pentane and cyclopentane (50:50 *v*/*v*; Sigma-Aldrich Corporation; Saint Louis, MO, USA). (0.5 pbw) distilled water.

**Figure 10:**
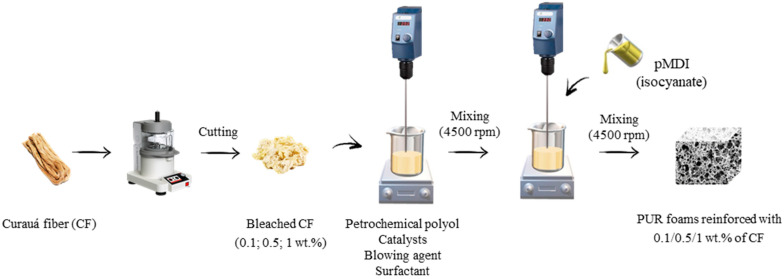
Flowchart for the manufacturing of rigid polyurethane foam composites.

Firstly, CF bleached pulp was fragmented by cutting for small (5 × 5 mm) squares then minced in a knife homogenizer (MPW-120) for 2 min. Rigid polyurethane foams (RPUF) were synthesized, based on previous reports [3,22]. Briefly, polyol, catalysts, surfactant, and blowing agents were mixed for 60 s at 4500 RPM (rotations per minute). The mixture was then incorporated with 0.1; 0.5 and 1 wt.% (named here as CF 0.1; CF 0.5 and CF 1, respectively) of bleached CF and, again, mechanically homogenized for 60 s at 4500 RPM. After that, MDI was added to the mixture and mixed again for 10–20 s at 4500 RPM. The obtained mixture was poured into an open, plastic box and allowed to grow freely in the vertical direction. All prepared foams were cured at room conditions (average temperature and moisture), for 24 h. After that, the samples were cut for further characterization. A schematic procedure of RPUF composite synthesis is presented in Figure 10.

Filled pre-polyol systems’ viscosities were evaluated using a Viscometer DVII+ (Brookfield, Dresden, Germany) in the function of a shear rate, according to ISO 2555 standard. The measurements were performed at room temperature.

RPUF cell structure was evaluated using a scanning electron microscope (SEM), JSM-5500 LV equipment (JEOL Ltd., Tokio, Japan). The samples were scanned parallel to the rise direction, at the accelerating voltage of 10 kV.

RPUF’s apparent density (10 samples for each group) was determined as the ratio of the foam’s mass to its volume, according to ISO 845 standard. Closed-cell content was evaluated based on PN-EN ISO 4590 standard, using the helium pycnometer AccuPyc 1340 with the Foam Pyc option (Micrometrics, Norcross, Norcross, GA, USA) in S.Z.T.K. ‘TAPS’—Maciej Kowalski Company (Lodz, Poland).

RPUF’s compressive strength (σ10%) (5 samples for each group) was evaluated according to ISO 844 standard, perpendicular and parallel to the foams’ rise direction, using a Zwick Z100 universal testing Machine (Zwick/Roell Group, Ulm, Germany, load cell of 2 kN, constant speed of 2 mm min^−1^). The measurements were performed up to 10% of samples’ deformation. Flexural strengths were also evaluated, according to ISO 178 standard. The measurements were performed using the same aforementioned universal testing machine and speed.

Mechanical reinforcement efficiency for foam strength (Γ_σ_), according to Equation (1), was calculated for compressive (parallel and perpendicular to the rise direction) and flexural properties, as in [31]. η_σ_ was considered as 1, due to the cell-wall stretching mechanism, presented for closed-cell foams and also due to the low anisotropy of the foams’ cells, as described in [59].

(1) $${\left(\Gamma_{C,F}\right)}_{\sigma }=\frac{{\left(\sigma_{C,F}\right)}_{c}}{{\left(\sigma_{C,F}\right)}_{f}}{\left(\frac{\rho_{f}}{\rho_{c}}\right)}^{n_{\sigma }}\frac{f_{\sigma }\left(R_{f}\right)}{f_{\sigma }\left(R_{C}\right)}$$

where: (σ_C,F_) is the compressive or flexural strength; ρ is the apparent density; f_σ_ (R_f_, R_c_) is a function that depends on the shape anisotropy ratio (R = cell length/cell width) and the property direction evaluation (parallel or perpendicular), and subscripts c and f represent the RPUF filled with CF and the neat RPUF, respectively.

Statistical analysis was performed for morphological and mechanical properties, using as a factor the wt.% of CF employed into the RPUF. Normality and homogeneity of each level were verified by Shapiro–Wilk and Levane tests, respectively. After that, one-way ANOVA and averaging tests were performed following the LSD Fischer procedure with 95% confidence.

RPUF’s dynamic mechanical analysis (DMA) was performed in an ARES rheometer (TA Instruments, New Castle, DE, USA). Measurements were carried out in the temperature range of 40–250 °C at a heating rate of 10 °C min^−1^, using a frequency of 1 Hz and the constant strain of 0.1%. To evaluate the influence of the content of CF used, the effectiveness of filler reinforcement constant, C, was calculated, as at [30,31], following Equation (2), from DMA tests:

(2) $$C=\left(\frac{{\left({E\prime }_{g}{/E\prime }_{r}\right)}_{\mathrm{Composite}}}{{\left({E\prime }_{g}{/E\prime }_{r}\right)}_{\mathrm{Matrix}}}\right)$$

where: E′_g_ and E′_r_ are the storage moduli related to the glassy and rubbery regions, respectively.

Thermogravimetric analysis (TGA) was performed for the studied RPUF, using Mettler Toledo thermogravimetric analyzer TGA/DSC1 (Columbus, OH, USA), at an argon heated atmosphere up to 600 °C. Decomposition temperatures (T_max1_, T_max2_, T_max3_, and residue, above 600 °C) were determined. RPUF’s thermal conductivities were measured using the heat flow meter apparatus Laser Comp 50 (TA Instruments, New Castle, DE, USA), with a 2.5 cm × 2.5 cm size heat flow transducer. The upper and lower plates of the HFMA instrument were set with a mean temperature of 25 °C.

RPUF’s flammability was evaluated by the cone calorimeter tests, according to ISO 5660 standard, in a S.Z.T.K. ‘TAPS’ equipment (Maciej Kowalski Company, Lodz, Poland). Each specimen with dimensions of 100 × 100 × 25 mm^3^ was wrapped with aluminum foil and burned at an external heat flux of 35 kW m^−2^. The parameters were recorded during the time (s).

## 4. Conclusions

Bleaching of Curauá fiber (CF) produced a fiber with lower impurities, dirty on its surface and higher aspect ratio, which enabled the use of the filler as a real reinforcement for the production of rigid polyurethane foams with high performance. Different amounts of CF (0.1, 0.5, and 1 wt.% in relation to the total RPUF mass) were investigated, aiming to evaluate the filler’s saturation on the PU matrix. At lower contents of CF (as those CF 0.1), improvements in mechanical properties were reported. However, those CF 0.5 presented the best mechanical performance, among the composites, due to the formation of a thinner and elongated cell structure. A ≈9% increment for all calculated mechanical strengths was reported for this content, compared to the neat RPUF.

Better dynamical mechanical and viscoelastic characteristics were also reported for CF 0.5. 19 °C improvements on T_g_, 23% higher values for E′_g_ and 80% for E′_r_, for those, compared to the neat foam. Better thermal stability was also achieved for those CF 0.5, compared to the neat foam, especially the temperatures related to the second and third steps of thermal decomposition. However, improvements in the fire resistance and flammability of the RPUF were not achieved, due to the high content of cellulose and hemicellulose on the CF chemical composition. This study focused on the use of CF, a high-performance vegetable Brazilian fiber, with low exploration on the current literature, for the manufacturing of cellular composite materials. The results presented in the current study, indicate that the use of CF, at an optimized content, enables the production of RPUF composites with improved physical-mechanical properties.

## Figures and Tables

**Table 1:** Rheological properties and processing times for the studied RPUF.

Sample	Temperature (°C)	Viscosity (mPa·s)	Cream Time (s)	Total Expansion Time (s)	Tack-Free Time (s)
Neat RPUF	154 ± 4	870 ± 10	42 ± 1	295 ± 5	358 ± 7
CF 0.1	155 ± 5	910 ± 12	44 ± 2	310 ± 6	360 ± 9
CF 0.5	152 ± 5	1110 ± 14	44 ± 2	325 ± 6	350 ± 7
CF 1	146 ± 3	1480 ± 15	46 ± 3	365 ± 5	345 ± 8

**Table 2:** Morphological characteristics for the studied RPUF composites.

Sample	Apparent Density (kg m^−3^) *	Cell Size (μm) *	R (μm) *
Neat RPUF	38.1^0.5^ B	480^1.0^ C	1.1^13.0^ A
CF 0.1	38.4^0.4^ C	485^0.8^ C	1.6^17.2^ B
CF 0.5	37.9^0.6^ B	470^1.1^ B	1.5^9.1^ B
CF 1	37.6^0.4^ A	450^1.1^ A	1.7^9.2^ B
F (*p* < 0.01)	28.7	31.6	7.4

* Coefficient of variance are the superscript values; different letters represent statistical differences between means for that property; F = test statistic.

**Table 3:** Mechanical properties and mechanical reinforcement efficiency for compression (ΓC) and flexural (ΓF) tests for the studied RPUF composites.

Sample	Compressive Strength (Parallel) (kPa) *	Compressive Strength (Perpendicular) (kPa) *	Flexural Strength (kPa) *	Maximum Elongation at Flexural Strength (%) *	ΓCparallel	ΓCperpendicular	ΓF
Neat RPUF	239.6^2.3^ B	140.4^2.9^ B	325.2^0.7^ B	11.2^2.7^ A			
CF 0.1	250.2^1.3^ C	145.8^2.3^ C	335.4^1.1^ C	10.8^2.8^ A	0.71	1.00	0.70
CF 0.5	260.4^1.2^ D	154.8^1.2^ D	354.8^0.7^ D	10.6^3.8^ A	0.79	1.09	0.79
CF 1	225.4^1.3^ A	120.2^3.0^ A	309.6^0.6^ A	13.5^2.2^ B	0.61	0.82	0.62
F (*p* < 0.01)	75.2	94.6	231.6	50.1			

* Values in superscript are the coefficient of variance; different letters represent statistical differences between means for that property; F = test statistic.

**Table 4:** Compilation of results from the DMA curves (Figure 5).

Sample	E’ Curves			Tan Delta Curves		
	E’g (kPa)	E’r (kPa)	C	Peak Height	FWHM (°C)	Tg (°C)
Neat RPUF	735	30	-	0.51	52.9	146
CF 0.1	887	36	1.00	0.53	58.3	162
CF 0.5	959	155	0.25	0.49	53.9	165
CF 1.0	644	105	0.24	0.46	66.0	144

E′_g_ at 50 °C; E′_r_ at 200 °C.

**Table 5:** Main results from thermogravimetric analysis (TGA) of the studied RPUF composites.

Sample	T_max_ (°C)			Residue (at 600 °C) (wt.%)
	1st Stage	2nd Stage	3rd Stage	
Neat RPUF	221	307	579	30.4
CF 0.1	215	309	583	29.8
CF 0.5	219	310	595	29.7
CF 1	229	311	593	29.6

**Table 6:** Main results from the cone calorimeter tests for the studied RPUF composites.

Sample	IT (s)	pHRR (kW m^−2^)	TSR (m^2^ m^−2^)	THR (MJ m^−2^)	COY (kg kg^−1^)	CO_2_Y (kg kg^−1^)	COY/CO_2_Y (-)	LOI (%)
Neat RPUF	4	257	1513	21.7	0.35	0.27	1.3	20.1
CF 0.1	4	264	1720	21.8	0.41	0.29	1.4	19.8
CF 0.5	3	285	1990	22.4	0.47	0.27	1.7	19.00
CF 1	3	290	1995	23.9	0.48	0.24	2.0	18.1

## Data Availability

Not applicable.
